# The Thebesian valve and coronary sinus in cardiac magnetic resonance

**DOI:** 10.1007/s10840-015-9994-3

**Published:** 2015-04-12

**Authors:** Rafal Mlynarski, Agnieszka Mlynarska, Maciej Haberka, Krzysztof S. Golba, Maciej Sosnowski

**Affiliations:** 1 Department of Electrocardiology, Upper Silesian Heart Centre, Ziolowa 45/47, 40-635 Katowice, Poland; 2 Department of Internal Nursing, School of Health Sciences in Katowice, Medical University of Silesia, Katowice, Poland; 3 Department of Cardiology, School of Health Sciences in Katowice, Medical University of Silesia, Katowice, Poland; 4 Department of Electrocardiology and Heart Failure, School of Health Sciences in Katowice, Medical University of Silesia, Katowice, Poland; 5 Unit for Noninvasive Cardiovascular Diagnostics, School of Medicine in Katowice, Medical University of Silesia, Katowice, Poland

**Keywords:** Coronary sinus, Thebesian valve, Cardiac magnetic resonance, CRT

## Abstract

**Purpose:**

There is no complex research exploring usefulness of cardiac magnetic resonance in the evaluation of the coronary sinus including Thebesian valve, which can be useful before selected electrophysiology procedures.

**Methods:**

One hundred twenty-two patients aged 49.2 ± 17.2 (42 women) were included in the study; 4 of them were excluded. A steady-state free-precession (SSFP) sequence was the basis of the visualization and analysis of the coronary sinus as well as Thebesian valve. In selected cases, dedicated coronary sinus sequences were created. All data were evaluated by experienced cardiac magnetic resonance investigators.

**Results:**

We were able to visualize the coronary sinus by using basic SSFP sequence in all patients, however in four cases in suboptimal quality. Average length of the coronary sinus was 39.73 ± 16.9 mm, average diameter was 9.81 ± 9.3 mm, and average angle of the entrance of the coronary sinus into the right atrium was 111.37 ± 13.8°. The Thebesian valve as the gate of the coronary sinus was found in 56 cases (45.9 %). In 21 patients (17.2 % of all), the valve was porous or almost totally covered the coronary sinus ostium, which can potentially create problems during CS cannulation.

**Conclusions:**

In most of the cases, it is possible to visualize and measure the coronary sinus using cardiac magnetic resonance with SSFP sequence. In selected cases, it is necessary to perform additional dedicated short sequences. Thebesian valve was visualized in almost 50 % of patients.

## Introduction

The *in vivo* anatomy of the coronary sinus including an analysis of Thebesian valve as well as target veins has been described in many papers [1–3]. The Thebesian valve is a caudal remnant of the embryonic sinoatrial valve. It is usually a semicircular fold of membrane in the right atrium at the orifice of the coronary sinus. It is on the posterior, inferior surface of the heart, medial to the inferior vena cava opening [1, 4]. Its role in normal physiology is not known, but some experts believe that it may prevent the regurgitation of blood into the sinus during the contraction of the atrium [5]. Unfortunately, experience from clinical practice suggests that the valve may pose difficulties during cannulation of the coronary sinus during cardiac resynchronization therapy (CRT). Therefore, studies on the anatomy of the Thebesian valve have potential practical implications [6, 7]. Visualization of the coronary venous system is an important element of cardiac resynchronization therapy as well as during ablation or even the delivery of stem cells into the heart [8]. The most common method of *in vivo* visualization to date is cardiac computed tomography (CT) in the consensus of experts [9]. A special scheme for the visualization of veins dedicated for cardiac resynchronization in cardiac CT was also presented by our team [10]. However, cardiac computed tomography has some serious disadvantages. Most of the patients who are qualified for CRT implantation are patients with advanced heart failure, for whom the administration of β-blockers during the examination is contraindicated. Many potential patients have also suffered renal failure, and therefore, the administration of a contrast agent is contraindicated.

There is an alternative—it is cardiac magnetic resonance (CMR). In this examination, it is possible to visualize the function of the heart as well as its anatomy in a noninvasive way using the next generation of CMR devices. An exact analysis of the ejection fraction in some patients for whom suboptimal images in the echo examination were obtained is recommended before qualification for an implantable cardioverter defibrillator (ICD). Using tagging function in some patients qualified for cardiac resynchronization may be also helpful in finding those who might respond well to the therapy. However, it is unclear whether it is possible to visualize the coronary venous system and Thebesian valve in CMR without creating special sequences to analyze the raw data.

The purposes of the study were to evaluate the usefulness of CMR in the evaluation of the coronary sinus including Thebesian valve, to design a methodology for fast coronary venous system visualization, and to evaluate the quality of CMR images.

## Methods

One hundred twenty-two patients aged 49.2 ± 17.2 (42 women) were included in this trial. Four of them were excluded due to significant anatomical anomalies of the heart found in the exam. Indications for an examination using cardiac magnetic resonance were typical, and they are presented in Table 1. Patients were excluded if they had impaired renal excretory function (calculated glomerular filtration rate below 30 mL/min/1.73 m) except during dialysis. Pregnant women or lactating women as well as patients with decompensated congestive heart failure who were unable to lie flat during an MRI or with a known allergy to gadolinium-based contrast agents were also excluded.

**Table 1:** Main indications for CMR in the patients included

Purpose	No. of cases	Percentage
Cardiomyopathy	49	40.2
Arrhythmogenic right ventricular dysplasia (ARVD)	17	13.9
Myocarditis	16	13.1
Ejection fraction evaluation	10	8.2
Overall	10	8.2
Cardiac neoplasm	9	7.4
Myocardial scar/viability	4	3.3
Sarcoidosis	3	2.5
Pulmonary hypertension	2	1.6
Anatomical abnormalities	2	1.6

Typical exclusion criteria for an MRI scan were the following:

- Cardiac pacemaker or implantable defibrillator implanted (acceptable if device and leads carry an MRI safety mark)
- Any other ferromagnetic implants that are not acceptable for cardiac MRI
- Any other implanted device (e.g., insulin pump, drug infusion device)

All magnetic resonance examinations were performed by using a GE Optima MR450w 1.5T with GEM Suite with a dedicated cardiac coil GE body 3D small cardiac. The GEM is an integrated system that combines high-density RF surface coils with software that permits high-quality images to be obtained. A highly homogeneous magnet with a 50 × 50 × 50-cm field of view to cover more anatomy is used in this equipment. Image acquisition parameters were as follows: slice thickness was 6–8 mm with a 1-mm interslice gap and field of view was typical 40 × 40 and matrix 192 × 192. Post-processing of images was performed by using a dedicated console.

Imaging protocol includes a steady-state free-precession cine imaging (SSFP; FIESTA/45) sequence which uses the T2 steady-state contrast mechanism for the evaluation of the cardiac anatomy, acquired in different standardized planes, including axial, 2-, 3-, and 4-chamber, and short-axis oblique planes covering the right atrium. These sequences provide images of fluid-filled structures in a very short time and were the basis of the visualization and analysis of the coronary sinus as well as Thebesian valve. More detailed assessment of the coronary venous vasculature was performed if there were difficulties with visualization of the coronary sinus ostium; in such cases, dedicated additional sequences were created.

All data were evaluated by two CMR investigators. An arbitrary scale of the quality of the images of the coronary venous system, which was adapted from the cardiac computed tomography scale of visualization, was introduced and used [10]. The highest grade on the scale was 5 and the lowest was 1, and the final score was the consensus of investigators.

**Fig. 1:**
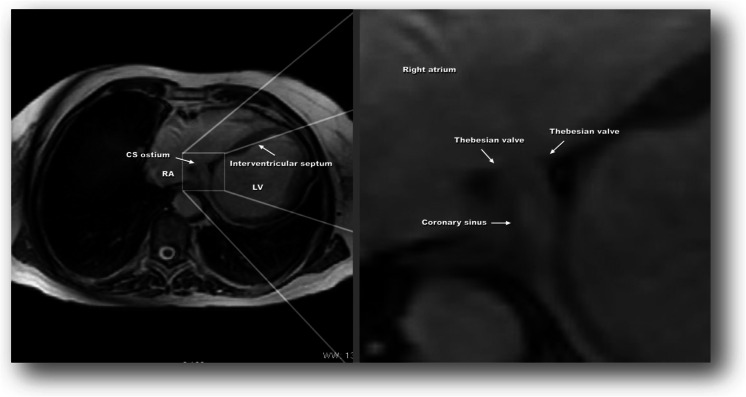
Example of the anatomy of the coronary sinus with a visible Thebesian valve in CMR/FIESTA (type C—Thebesian valve is built from two separate parts; there is a gap between parts). *RA* right atrium, *LV* left ventricle, *CS* coronary sinus

An example of anatomy of the coronary sinus with visible Thebesian valve in CMR/fast imaging employing steady-state acquisition (FIESTA) is presented in Fig. 1. The nomenclature for the Thebesian valve was based on the classifications of the Thebesian valve in cardiac computed tomography that were proposed and published by the authors [1]. This nomenclature is presented in Table 2.

**Table 2:** Proposed nomenclature for the types of Thebesian valves adapted from cardiac computed tomography [1]

Type of Thebesian valve	Description
A1	The semilunar membrane is visible from the atrium wall. The membrane covers less than 50 % of CS ostium
A2	The semilunar membrane is visible from the atrium wall. The membrane covers more than 50 % of CS ostium
B1	The semilunar membrane is visible from the interatrial septum. The membrane covers less than 50 % of CS ostium
B2	The semilunar membrane is visible from the interatrial septum. The membrane covers more than 50 % of CS ostium
C	Thebesian valve is built from two separate parts. There is a gap between parts
D	The almost whole CS ostium is covered by a membrane

## Results

The hemodynamic measurements of the patients included that were obtained using cardiac magnetic resonance are presented in Table 3.

**Table 3:** Average values of the main cardiac function parameters for the patients included

	Average value	±SD
Ejection fraction (%)	47.56	±14.9
Stroke volume (mL)	71.74	±22.3
End diastolic volume (mL)	168.73	±82.2
End systolic volume (mL)	97.42	±74.5
Peak filling rate (mL)	324.18	±125.0
Peak ejection rate (mL)	384.76	±138.4
Cardiac output (L/min)	4.58	±1.5

We were able to visualize the coronary sinus by using steady-state free-precession (SSFP) scans in all patients, however in four cases in suboptimal quality. Using this sequence, the average length of the coronary sinus that was visualized was 39.73 ± 16.9 mm. The average diameter of the coronary sinus ostium was 9.81 ± 9.3 mm. Average diameter of the ostium in patients with a valve present was 11.9 ± 17.9 and without Thebesian valve was 8.9 ± 0.4; *p* = 0.429 NS. The average angle of the entrance of the coronary sinus into the right atrium was 111.37 ± 13.8°. We were able to visualize the proximal parts of posterolateral veins in 66 (54.1 %) patients.

The quality of the visualization of the coronary sinus in most cases (109 cases; 89.2 %) was acceptable to perform a clinical analysis (mean score obtained was 4, range 3–5). In 24 (19.7 %) patients, we obtained optimal quality (score 5) of visualization. Analysis for the patients with quality score 1–2 (10.8 %) made it difficult to draw any conclusions. Average quality of visualization was 3.55 ± 0.9.

**Fig. 2:**
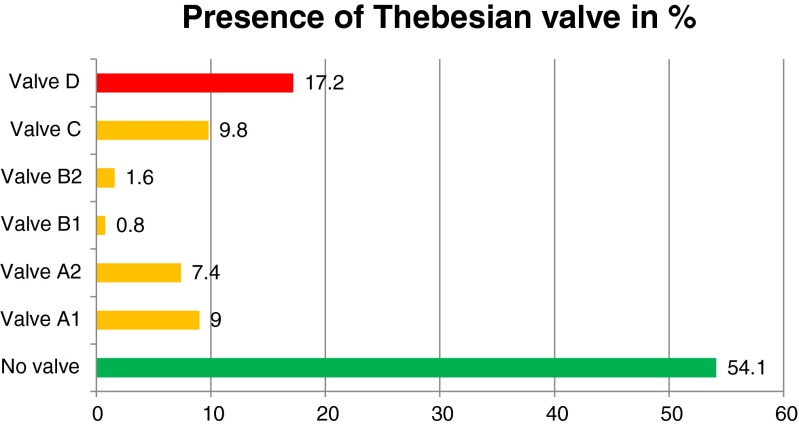
Graphic distribution of different types of Thebesian valves

**Fig. 3:**
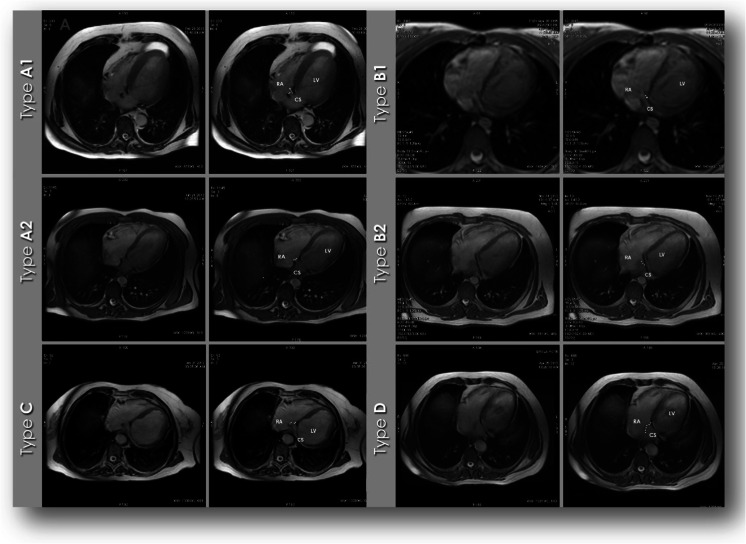
Examples of various types of Thebesian valves in CMR/FIESTA. *RA* right atrium, *LV* left ventricle, *CS* coronary sinus

**Fig. 4:**
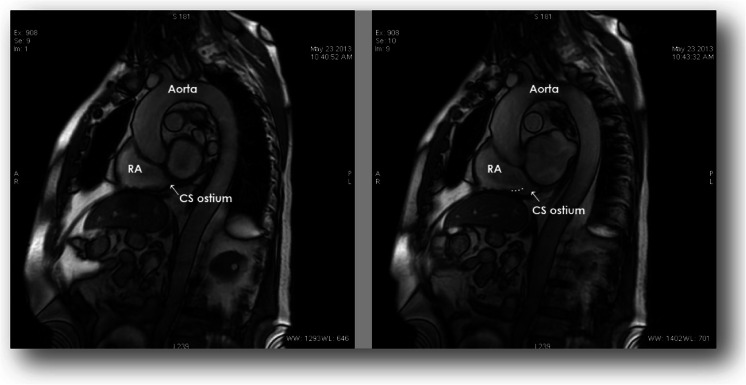
Additional sequence of the visualization of the coronary sinus ostium to the right atrium if the basic visualization (FIESTA) was not acceptable diagnostically. *RA* right atrium, *CS* coronary sinus

The Thebesian valve as the gate of coronary sinus was found in the 56 cases (45.9 % included patients). Graphic distribution of different types of Thebesian valves is presented in Fig. 2. The most frequent variant (21 cases; 17.2 % of all included) was D in which a significant part of the sinus ostium is covered by the valve. What is most important is that in each case in which the Thebesian valve was found, we were able to describe its clinical characteristics. Examples of Thebesian valves are presented in Fig. 3. If visualization SSFP was not acceptable diagnostically, an additional visualization sequence of the coronary sinus ostium to the right atrium was performed. An example of such a sequence is presented in Fig. 4.

## Discussion

**Fig. 5:**
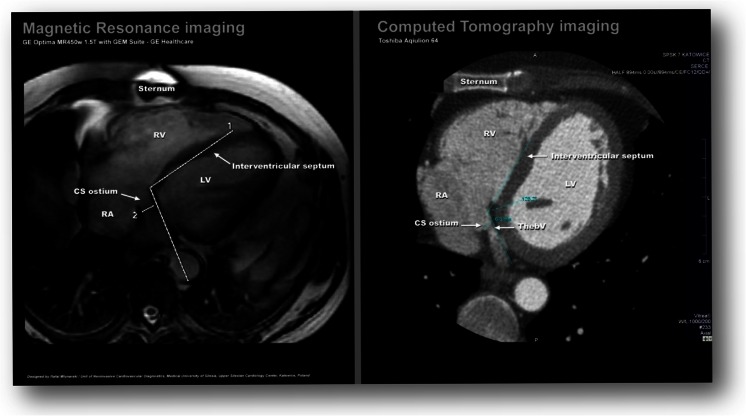
Graphic comparison of the visualization of the coronary sinus ostium in computed tomography as well as CMR. *RA* right atrium, *LV* left ventricle, *CS* coronary sinus

The anatomy of the coronary sinus was well recognized *in vivo* in cardiac computed tomography [1, 10, 11]. The popularity of cardiac magnetic resonance has increased, and this examination has some advantages, especially for patients for whom no contrast agent can be used and those who cannot be exposed to radiation. It is important to remember that the visualization of vessels is not the strongest aspect of CMR. A comparison of cardiac computed tomography from our image library with CMR from this research is presented in Fig. 5. It can be seen that the images are comparable. In our CT image database of the coronary venous system, there is 46∼55 % visualization of the Thebesian valve [1]. Those results are almost equal with the results presented in this study (45.9 %).

After analyzing the literature on the subject, we are aware that there is not one common standard for the visualization of coronary veins despite the fact that a few papers have been published. When we prepared the methodology for this paper, one of the aims was to simplify and check whether it is possible to analyze images retrospectively. As a result, we first tried to use the sequence that is always performed during cardiac magnetic resonance—FIESTA. The FIESTA sequence uses a T2 steady-state contrast mechanism to provide high SNR images with a strong signal from fluid-filled tissues while suppressing background tissue in order to produce a contrast and anatomic detail of small structures. In addition, the ultrashort repetition time (RT) and echo time (TE) permit extremely short acquisition times—shorter than fast spin echo (FSE)—and the images can be post-processed using MIP, volume rendering, or 3D navigator techniques.

As was mentioned earlier, our paper is not the first that describes the visualization of coronary veins using CMR. However, none of the papers cited below analyzed the Thebesian valve. Younger et al. in 2009 examined 31 cardiac CMR studies in 3D reconstructions with gadolinium [12]. The authors confirmed that the cardiac venous system was visualized in all of the patients. They also visualized the anterior lateral and posterior veins. They documented myocardial infarction on late gadolinium enhancement (LGE) images in five patients. The authors concluded that the coronary venous anatomy can be reliably demonstrated using a comprehensive CMR protocol and a standard extracellular contrast agent. Like us, they think that a combination of coronary venous anatomy imaging, assessment of ventricular function, and LGE may be useful in the management of patients with LV dysfunction who are being considered for CRT.

Chiribiri et al. [13] examined 31 participants in CMR using whole-heart imaging and intravascular contrast agents, and Younger et al. [12] used 3D reconstructions along with standard orthogonal planes in all of the coronary sinuses and great cardiac veins with relations to the mitral valve and LCx. Both authors visualized the coronary sinus in all of the participants. The authors concluded that cardiovascular magnetic resonance might provide important information for the selection of candidates for these procedures. Similarly, Ibrahim et al. reported possibility of visualization of the coronary sinus and great cardiac, posterior interventricular, and anterior interventricular veins in 100 % of patients by both MRI and MDCT [14]. Detection of the posterior vein of the left ventricle and the left marginal vein by MRI was reported as 97 and 81 %, respectively.

We agree that whole-heart 3D reconstructions are more useful to recognize the anatomy of the coronary venous system; however, in order to add clinical value, an analysis of the Thebesian valve and of its types (if present) should be always performed.

## Conclusions

In most cases, it is possible to visualize and measure the coronary sinus using cardiac magnetic resonance with SSFP sequence. In selected cases, it is necessary to perform additional, dedicated short sequences. Thebesian valve was visualized in half of the patients, which may provide a way to recognize problems for CRT implantation and other procedures.

## Limitations of study

We agree that visualization of the coronary sinus and Thebesian valve may not be recommended for selection of patient for CRT due to limited data actually available. However, the decision of a surgeon to implant a CRT may be affected by these additional imaging data. In our opinion, cardiac magnetic resonance may create a road to visualize Thebesian valve and to visualize potential obstruction of the coronary sinus ostium. The authors believe that imaging data are mostly created to support surgeons in the decision-making process and when choosing the most optimal equipment. Another technical disadvantage of cardiac magnetic resonance is the lack of the possibility to perform another reconstruction after finishing the examination (except 3D whole-heart imaging if available).
